# Supplementary material for: Movement-assisted localization from acoustic telemetry data
Source: Mov Ecol. 2020 Jun 30;8:15. doi: 10.1186/s40462-020-00199-6 (PMC7327795; doi:10.1186/s40462-020-00199-6)
Supplement: Supplementary file 2 — Additional file 2 Animation of the latent trajectories for two individuals. Animations denote detection and non-detection locations (filled and open red dots, respectively; see Fig. 2 for additional details) and model- and occasion-specific posterior localization estimates (black points). Modeling approaches include (a) the independent localization model and (b - d) three forms of movement-assisted localization: (b) detection occasions only, (c) assuming all time intervals are known, or (d) modeling the unknown time intervals as random variables. Receiver array is denoted by blue x’s and receivers that recorded a detection at each time step are highlighted in red. [file 40462_2020_199_MOESM2_ESM.pptx]

## Slide 1
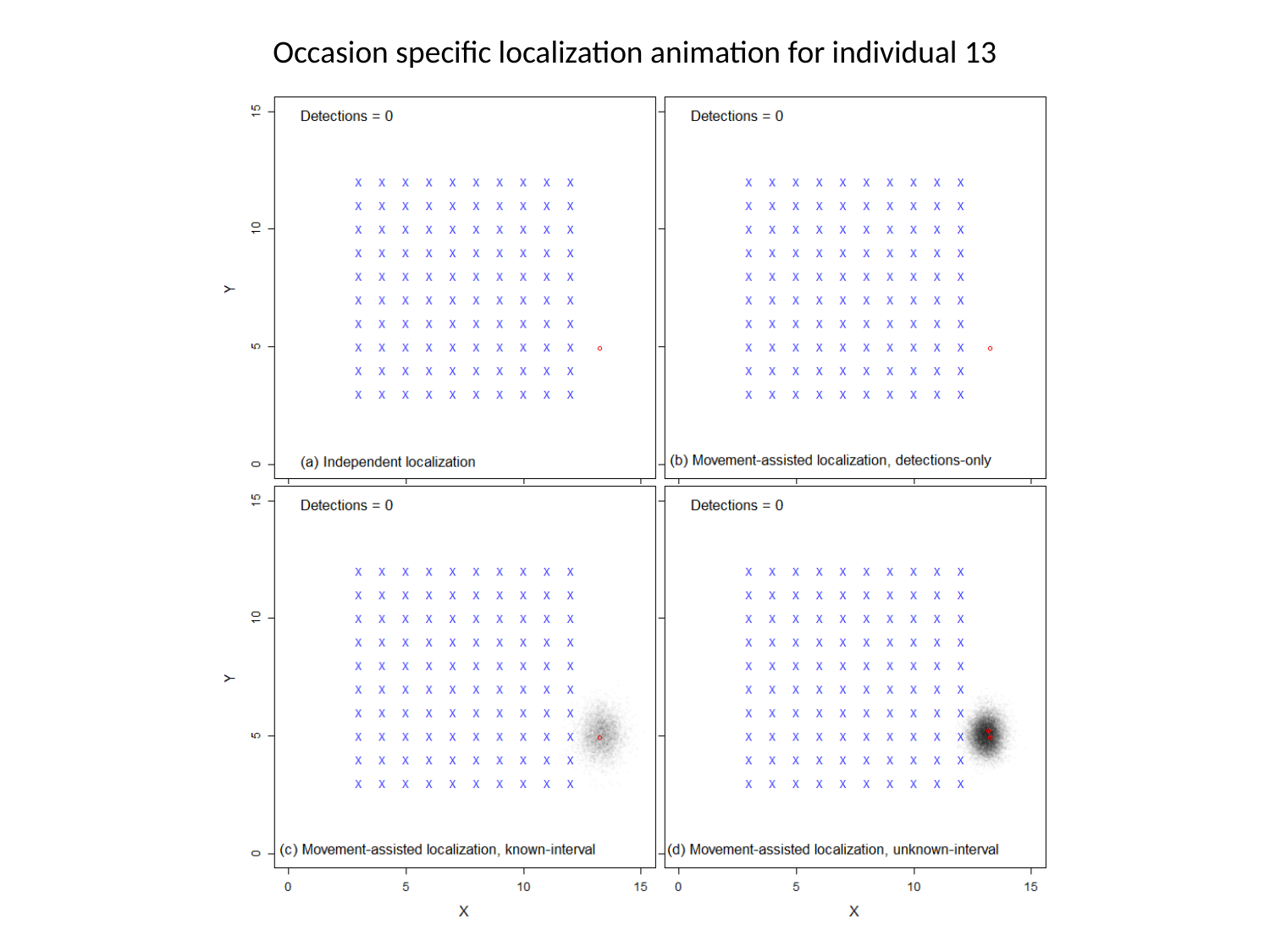

Occasion specific localization animation for individual 13

## Slide 2
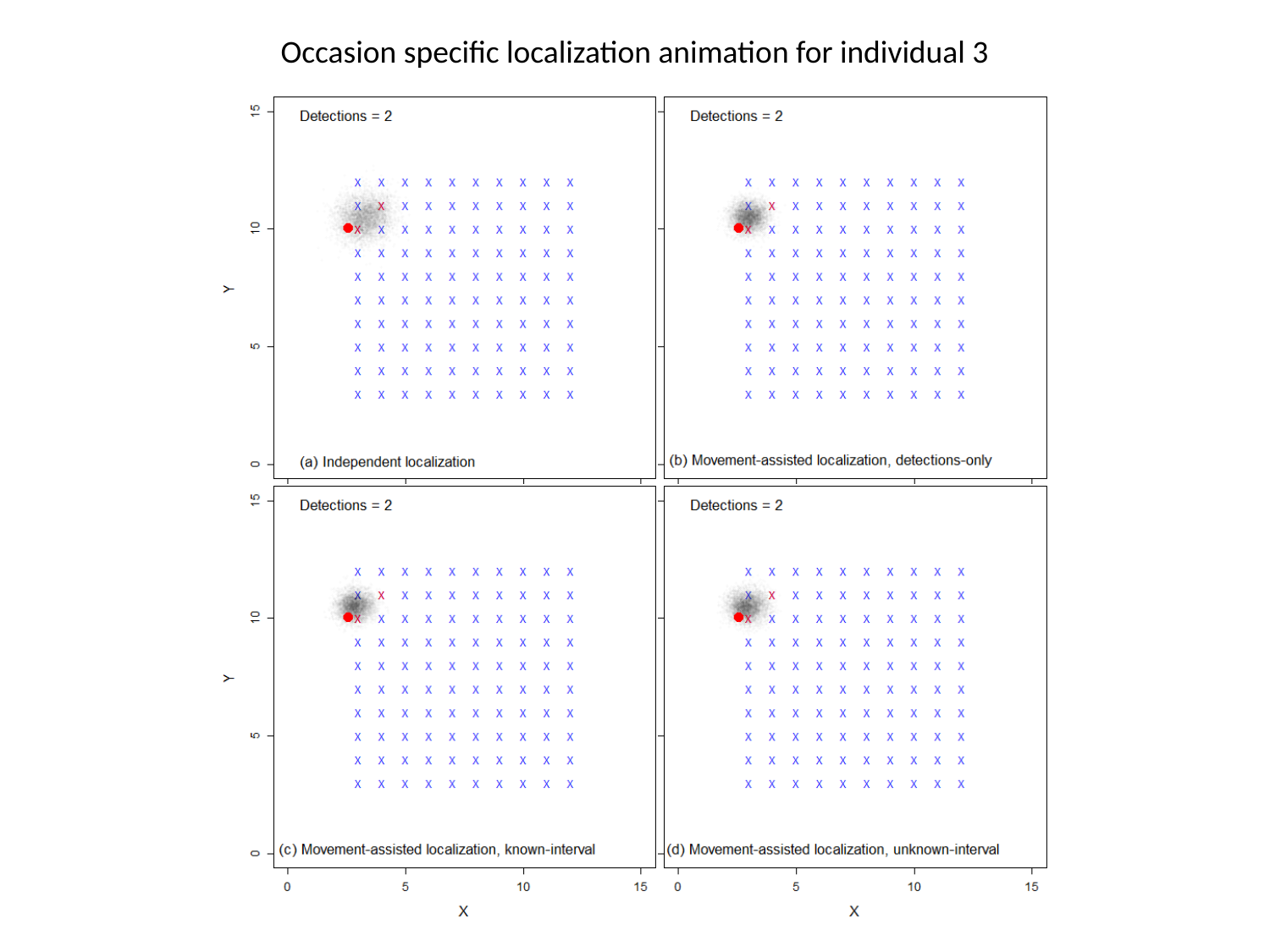

Occasion specific localization animation for individual 3
